# Adenocarcinoma of the caecum metastatic to the bladder: an unusual cause of haematuria

**DOI:** 10.1186/1471-2490-6-29

**Published:** 2006-10-14

**Authors:** Benjamin R Grey, Laurence Clarke, Satish B Maddineni, Roger Hunt, Richard J Brough

**Affiliations:** 1 Department of Urology, Stepping Hill Hospital, Stockport, UK; 2 Department of Histopathology, Stepping Hill Hospital, Stockport, UK

## Abstract

**Background:**

Primary malignancies of colorectal origin can metastasise to the bladder. Reports are however extremely rare, particularly from the caecum.

**Case report:**

The report describes the case of a 45-year old male with Duke's B caecal carcinoma treated with a laparoscopically-assisted right hemicolectomy and adjuvant 5-Fluorouracil chemotherapy. Subsequently, a metastatic lesion to the bladder was demonstrated and successfully excised by partial cystectomy.

**Conclusion:**

In order that optimal therapeutic options can be determined, it is important for clinicians to distinguish between primary disease of the bladder and other causes of haematuria. Various immunohistochemical techniques attempt to differentiate primary adenocarcinoma of the bladder from secondary colorectal adenocarcinoma. Suspicion of metastatic disease must be raised when histologically unusual bladder tumours are identified.

## Background

The vast majority of bladder tumours are primary transitional cell carcinomas. Primary adenocarcinomas of the bladder are rarer but well recognized. Secondary neoplastic growths of the bladder account for approximately 2% of bladder malignancies [1]. Bladder involvement in patients with primary colorectal adenocarcinomas is most likely to be secondary to direct invasion from the adjacent recto-sigmoid. Isolated distant metastases to the bladder from colorectal primary disease have very rarely been reported [2-5]. This case presents a case of frank haematuria related to a bladder metastasis from a mucinous adenocarcinoma of the caecum.

## Case presentation

A 45-year-old man presented with vague abdominal pain associated with a palpable mass. An ultrasound scan demonstrated a mass in the right iliac fossa but no evidence of hepatic metastases or lymphadenopathy. A plain X-ray film of the chest demonstrated no abnormality. A subsequent barium enema suggested a caecal carcinoma and he was subsequently treated for a pT3 N0 M0 (Dukes B) moderately differentiated mucinous adenocarcinoma of the caecum in July 2002 with a laparoscopically-assisted right hemicolectomy. A complete oncological resection was achieved with clear surgical resection margins of at least 4 centimetres. Fourteen lymph nodes were sampled but no metastatic disease was identified. In concordance with his high risk for microsatellite instability, he underwent 30 sessions of weekly adjuvant 5-Fluorouracil (5-FU) chemotherapy with minimal co-morbidity. Eight months post-operatively he was referred to the Urology Department with a 4-month history of irritative lower urinary tract symptoms that had culminated with a short history of frank haematuria.

**Figure 1:**
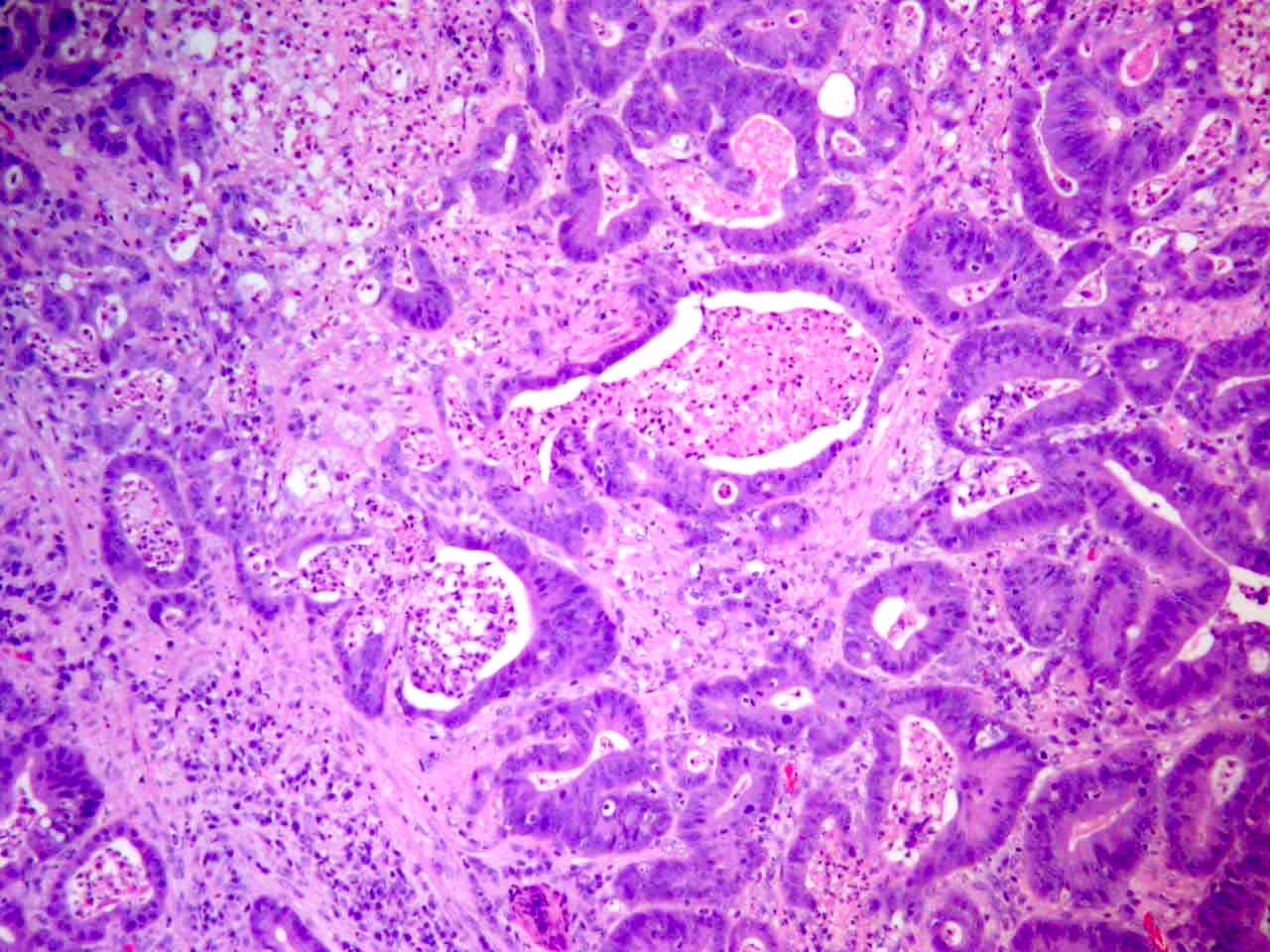
Moderately differentiated adenocarcinoma with extensive luminal necrosis.

An initial flexible cystoscopy revealed a solid-looking 2 cm growth on the posterior bladder wall. A subsequent intravenous urogram demonstrated normal upper tracts. He underwent trans-urethral resection of the bladder tumour (TURBT) through to deep muscle. Histology of the TURBT specimen revealed a moderately differentiated adenocarcinoma with the following immunoprofile: CK20 and CDX2 +ve, but CK7 -ve. This tissue contained no evidence of extensive cystitis cystica glandularis, intestinal metaplasia or any in-situ component, as may be seen in a primary bladder adenocarcinoma. Comparison was made with the original caecal pathology. Morphologically and immunohistochemically these specimens were identical, suggesting bladder metastases from the primary caecal malignancy (Figure 1). A computed tomography (CT) scan at this time revealed no sign of further metastatic disease. The Oncology Department arranged for him to receive CT-staged 3-D conformal multi-fractionated radiotherapy to a total dose of 56 Gray (Gy).

**Figure 2:**
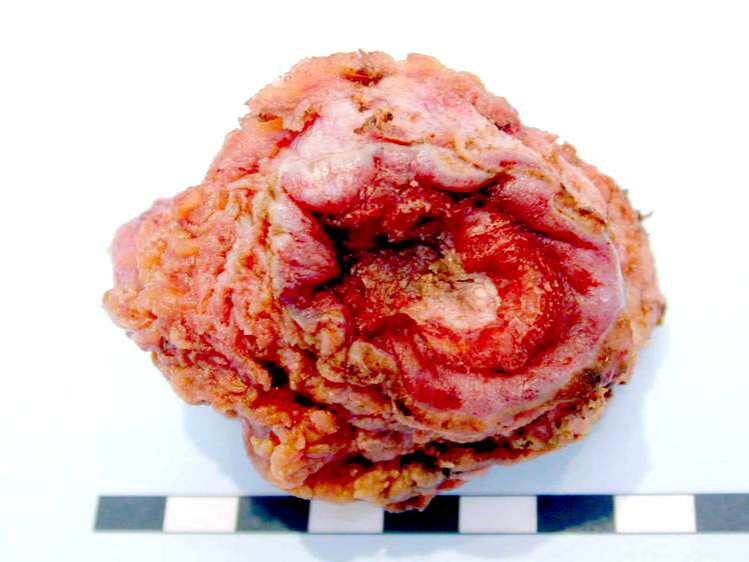
Luminal surface of partial cystectomy specimen with deposit of metastatic carcinoma.

**Figure 3:**
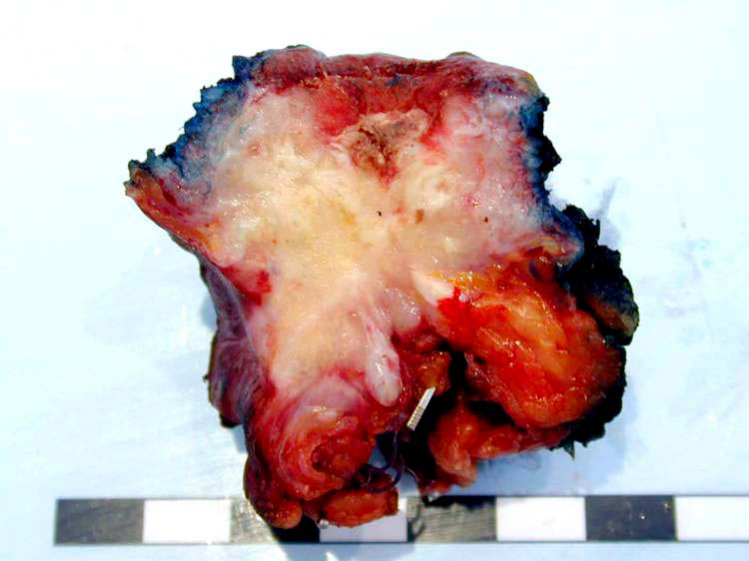
Sagittal slice of partial cystectomy specimen revealing superficial carcinoma.

Ten months following the original TURBT, recurrent bladder tumour was found in the same position at check cystoscopy. This was confirmed to be recurrent moderately differentiated intestinal-type adenocarcinoma. Magnetic Resonance scanning revealed a mass involving the right antero-lateral bladder wall and dome extending to the perivesical fat and the right vas deferens. Review of the scans at the Uroradiology multidisciplinary team meeting suggested the lesion to actually be stage pT2b with associated post-TURBT perivesical inflammatory changes. There was no evidence of lymphadenopathy or metastatic disease. Furthermore neither recurrent colon tumour nor any direct involvement between bowel and bladder were identified. Subsequently he was offered and underwent a partial cystectomy (Figures 2 and 3). There was no evidence of recurrent disease at twelve-month review.

## Discussion

Distant metastases to the bladder have been reported in patients with primary stomach [2,6], skin [2,7,8], breast [2,9-11], lung [2], pancreatic [2,12], uterine [2,13] and oesophageal malignancies [14]. Caecal primaries are anatomically less likely to invade the bladder than neoplasms of the rectosigmoid. This rare case provides evidence consistent with distant metastases to the bladder which were subsequently treated surgically with clear resection margins (Figure 3).

A correct distinction between primary adenocarcinoma of the bladder and secondary colorectal adenocarcinoma is important for staging of disease, determining appropriate treatment and ultimate prognosis [15]. In this case, the patient had already received treatment for a caecal tumour. If however the bladder metastasis was the presenting feature, it would be important to identify and evaluate the underlying primary in the colorectum. Immunohistochemical similarities between primary adenocarcinoma and secondary colorectal adenocarcinoma of the bladder however make this task difficult [15,16].

**Figure 4:**
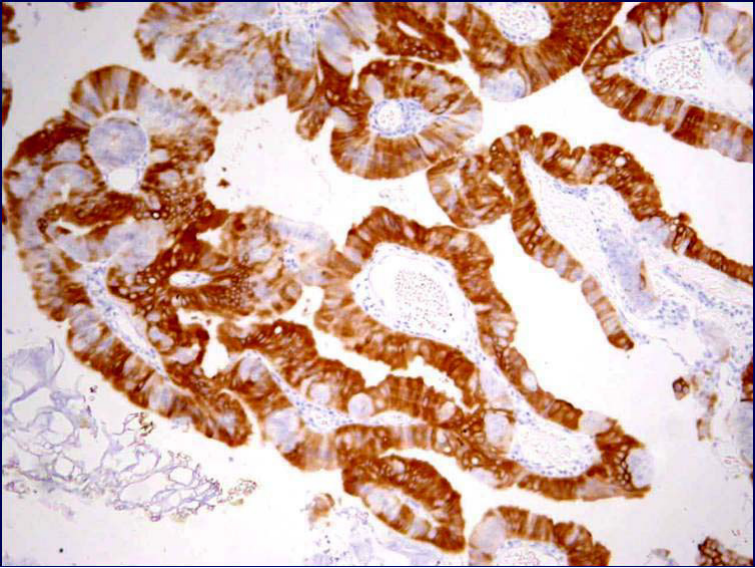
Strong cytoplasmic and membranous CK20 positivity in metastatic colorectal carcinoma.

As in this case, immunostains CK7 and CK20 are commonly used to aid diagnosis, with most lesions of colorectal origin having a CK7 negative and CK20 positive profile (Figure 4). The study by Wang et al. in 2001 reinforced previously published results [17], suggesting stains for CK7 and CK20 were positive in greater than one half of primary bladder adenocarcinoma tissue samples. The authors however concluded that combined staining for CK7 and CK20 alone did not provide sufficient specificity for accurate differentiation between the two clinical scenarios. The investigators discussed the failure of the tumour suppressor adenomatous polyposis coli (APC) gene in colorectal tumorigenesis and the consequent rise in the cell degradation product β-catenin. Nuclear staining of metastatic colonic carcinoma with β-catenin aids in distinguishing primary from metastatic adenocarcinomas with nuclear positivity in 81% of metastatic tumours compared to less than 1% of primary carcinomas [15]. The study by Wang et al. also suggested that thrombomodulin expression as a marker of primary bladder carcinoma adds to the specificity of the CK7 and CK20 profile. 59% of primary bladder adenocarcinomas demonstrated significant expression of membranous thrombomodulin compared to 0% of colonic adenocarcinomas [15]. CDX2 is a homeobox transcription factor that regulates the differentiation of intestinal epithelial cells. As in this case, it has been shown to be of diagnostic use in aiding distinction between primary adenocarcinoma of the bladder and secondary colorectal carcinoma [18]. Tamboli et al. also suggested the use of stains for Villin and concluded that their use, in addition to CK7 and CK20, allowed urothelial carcinoma with glandular differentiation to be distinguished from secondary colonic carcinoma [19]. The available evidence suggests that an immunohistochemical panel of CK7, CK20, CDX2, beta-catenin, thrombomodulin and Villin may best distinguish primary bladder adenocarcinomas from metastases. However, it should be noted that in total the current literature contains relatively few cases of primary bladder adenocarcinoma that have been evaluated immunohistochemically and further work in this field is needed before a definitive panel that enables discrimination between primary and metastatic bladder adenocarcinomas can be established.

## Conclusion

This case highlights the rare complication of a distant bladder metastasis from a caecal carcinoma. This was successfully treated with a surgical resection and the patient had not suffered recurrence after twelve months of follow-up. Suspicion of metastatic disease must be considered when histologically unusual bladder tumours are identified. In the absence of pre-existing malignant disease immunohistochemical panels of antibodies are often required to try and identify the focus of primary disease thus allowing appropriate treatment regimen to be formulated.

## Abbreviations

TURBT: Transurethral resection of bladder tumour

## Competing interests

The author(s) declare that they have no competing interests.

## Authors' contributions

RJB was the lead clinician responsible for the patient's care, performed the partial cystectomy and along with SBM and RH conceived the idea for the report. RH carried out the pathological and immunohistochemical analysis of the specimens. Furthermore, RH provided appraisal of the pathological discussion. BRG, LC and SBM were also members of the surgical team, reviewed the case notes and drafted the manuscript. All authors read, appraised and approved the final manuscript.

## Pre-publication history

The pre-publication history for this paper can be accessed here:
